# What Is New in the Field of Industrial Wastes Conversion into Polyhydroxyalkanoates by Bacteria?

**DOI:** 10.3390/polym13111731

**Published:** 2021-05-26

**Authors:** Paulina Marciniak, Justyna Możejko-Ciesielska

**Affiliations:** Department of Microbiology and Mycology, Faculty of Biology and Biotechnology, University of Warmia and Mazury in Olsztyn, Oczapowskiego 1A, 10719 Olsztyn, Poland; paulina.marciniak@uwm.edu.pl

**Keywords:** polyhydroxyalkanoates, waste feedstocks, pure bacteria culture

## Abstract

The rising global consumption and industrialization has resulted in increased food processing demand. Food industry generates a tremendous amount of waste which causes serious environmental issues. These problems have forced us to create strategies that will help to reduce the volume of waste and the contamination to the environment. Waste from food industries has great potential as substrates for value-added bioproducts. Among them, polyhydroxyalkanaotes (PHAs) have received considerable attention in recent years due to their comparable characteristics to common plastics. These biodegradable polyesters are produced by microorganisms during fermentation processes utilizing various carbon sources. Scale-up of PHA production is limited due to the cost of the carbon source metabolized by the microorganisms. Therefore, there is a growing need for the development of novel microbial processes using inexpensive carbon sources. Such substrates could be waste generated by the food industry and food service. The use of industrial waste streams for PHAs biosynthesis could transform PHA production into cheaper and more environmentally friendly bioprocess. This review collates in detail recent developments in the biosynthesis of various types of PHAs produced using waste derived from agrofood industries. Challenges associated with this production bioprocess were described, and new ways to overcome them were proposed.

## 1. Introduction

Synthetic polymers are distinguished by such features as a high level of strength, durability and lightness. Moreover, they could be easily chemically modified which makes them versatile and innovative materials used in countless products from nearly all industrial sectors. However, production of petrochemical plastics has led to environmental problems because they are accumulated in the most varied environments and are not easily degradable. Worldwide production of plastics is still increasing reaching 359 million tons in 2018, so 155 million tons more compared to 2002 [1]. In addition, in spite of many efforts to increase the amount of plastic waste sent to recycling, only 32.5% were recycled in Europe in 2018. The remaining plastics are discarded in the landfills, left in the open dumps or in the natural environment. During their decomposition process, microplastics (MPs) are formed which have negative impacts on the environment that have become a global concern. Particles of microplastics can easily enter into the interior of the body by water or food and be transferred through the food chain to other organisms [2]. Therefore, the synthetic polymers need to be substituted by their ecofriendly alternatives. Furthermore, biobased substitutes for petrochemical polymers that are generated in microbial processes using renewable resources can reduce emissions of greenhouse gases, and the materials produced based on the biopolymers are easier to recycle to the virgin polymer and, at the end of their useful life, biodegrade in the environment [3].

Among the various groups of biopolymers, polyhydroxyalkanoates (PHAs) can be used to replace a wide variety of traditional plastics. They are synthesized and accumulated by microorganisms in their cytoplasm in the form of granules [4]. The synthesis of PHAs in bacterial cells takes place under unbalanced growth conditions when carbon source is present in excess and nutrients such as nitrogen or phosphorus are in deficiency [5]. Due to biodegradability and biocompatibility, PHAs have gained much attention of industry in recent years. It is predicted that the bioplastic industry will be a key player on the materials market in the future. The production of PHAs from commercial manufacturers reached 2.05 million tons in 2017. It is believed that the global market of PHAs will grow up to 98 million tons by 2024 [6]. Many companies have been set up to commercialize PHAs as environmentally friendly bioplastics using renewable resources as primary or secondary feedstocks (Table 1). However, the deployment of PHAs into the global market is still in its early stages. Because of their high production costs, PHAs wide commercialization and industrialization is limited. Therefore, there is a need to employ cheaper substrates that will be used for bacterial growth and will support PHAs synthesis and accumulation. In order to decrease costs and make PHAs production bioprocess more economically feasible, much research has been conducted using industrial waste streams for microbial fermentation processes. On the other hand, the use of waste carbon sources for microbial-derived PHAs production could reduce waste disposable costs [7].

Due to the rising global population and, as a consequence, increased food processing demand, a tremendous amount of food waste is generated, causing serious environmental problems. Recently, great efforts are made to convert food waste into value-added products such as biohydrogen, volatile fatty acids [15], bioactive compounds [16] or biopolymers [17]. Despite various types of waste that can be used as substrates for PHAs production, recently more attention has been focused on the agri-food wastes which have a high rate of production worldwide. These wastes seem to be potential raw materials for bacterial fermentation to produce value-added PHAs being a good strategy not only for the promotion of the waste’s recovery but also the development of desirable bioproducts. 

The aim of this review is to discuss current knowledge on the use of agro-industrial wastes as carbon sources for PHAs production. Moreover, the challenges and prospects of using them in the fermentation processes for PHAs industrial scale production will be presented. The recent review published by Kumar et al. [18] on the similar topic was focused on the economy of PHAs production by bacteria, their extraction and applications, whereas the presented article described comprehensively the latest progress in PHAs production using wild-type and recombinant bacteria. 

## 2. PHAs Characterization and Biosynthesis

The chain of PHAs consists of from 600 to 35,000 hydroxy fatty acid monomer units. It is polyester in which the side chain R group is usually a saturated alkyl group. Depending on the total number of carbon atoms in a monomer, PHAs are divided into three groups: short-chain-length PHAs (PHA_SCL_) which contain from 3 to 5 carbon atoms, medium-chain-length PHAs (PHA_MCL_) with 6–14 carbon atoms in each unit and long-chain-length PHAs (PHA_LCL_) which contain more than 15 carbon atoms [5]. PHAs are characterized by several useful traits. PHA_SCL_ like poly(3-hydroxybutyrate) [(P(3HB)] is a highly crystalline polymer with high brittleness, high melting temperature and glass transition temperature, and with poor elastic properties. They are produced by many bacterial species such as *Bacillus megaterium*, *Cupriavidus necator*, *Aeromonas hydrophila*. PHA_MCL_ like poly(3-hydroxyhexanoate) [P(3HHx)] possess more desirable properties. They have low degree of crystallinity, low tensile strength, high elongation to break, low melting temperature and high elasticity. PHA_MCL_ are synthesized mainly by *Pseudomonas* species. PHA_LCL_ like poly(3-hydroxyhexadecanoate) [(P(3HHxD)] is uncommon and knowledge about its properties and potential applications is still limited. The most important feature of all types of PHAs is their ability to be degraded after being discarded. The degradation of these biopolymers lasts up to one year and is a result of the action of extracellular enzymes called PHA depolymerases synthesized by some bacteria and fungi [19]. It is well known that the efficiency of their decomposition is dependent on several factors, e.g., activity of microorganisms, environmental conditions, temperature and PHAs properties like molecular weight [20]. Moreover, unlike synthetic polymers like polypropylene, PHAs are hydrophobic, nontoxic, insoluble in water and more resistant to UV radiation [21]. These properties enable their wide range of application (Table 2).

Recent advances in the biodegradability of PHAs have been comprehensively described by Meereboer et al. [22]. It is known that PHA-degrading bacteria could be divided into two groups: microorganisms that physically degrade PHAs and those which metabolize the by-products of PHAs degradation. PHAs are considered the most readily degradable biopolymer both in aerobic and anaerobic conditions compared to other biopolymers such as polylactide, polybutylene succinate or polybutyrate adipate terephthalate. However, taking into account the industrial applications based on PHA monomers the addition of additives or other biodegradable/nonbiodegradable polymers is needed to improve their properties. It was reported that antifouling agents or plasticizers influence negatively on the PHA biodegradation process [23]. On the other hand, incorporation of natural fibers could improve the rate of PHA biodegradation [24]. Therefore, the research in the area of biodegradation processes of PHA biocomposites should be conducted to understand deeper a biodegradable nature of PHAs. 

It is estimated that up to 150 bacterial genera are capable of producing PHAs [25]. PHAs-producing bacteria that are able to grow on waste feedstocks include those commonly found in the environment, e.g., in soil [26,27]. Recent studies indicated the potential of marine bacteria to PHAs synthesis, among them the special attention has gained *Halomonas* sp., *Salinivibrio* sp. [28,29,30,31].

**Figure 1:**
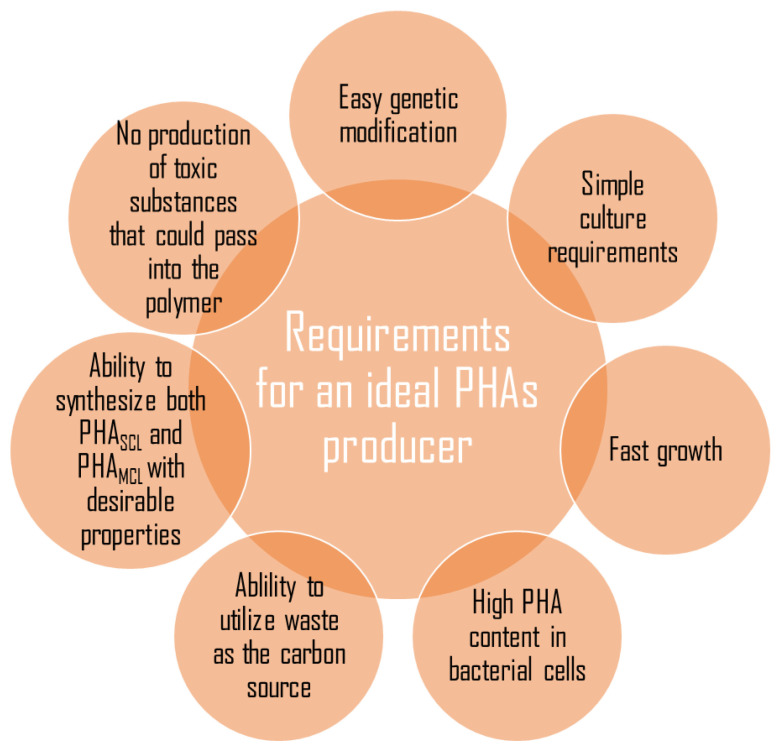
Characteristics of an ideal microorganism that could be used for a large scale production of polyhydroxyalkanoates.

PHA synthases are the key enzymes involved in the polymerization of PHAs using the (R)-3-hydroxyacyl-CoA as a substrate. They are divided into four class depending on their subunit composition, amino acid sequence, and substrate specificity. PHA synthases belong to group I, III, and IV are found to polymerize short-chain-length monomers, and group II medium-chain-length monomers. Group I and group II PHA synthases contain a single PhaC subunit with molecular mass between 61 and 73 kDa; they were found in *Cupriavidus necator* and *Pseudomonas* species, respectively. Group III and IV synthases consist of two subunits. Group III synthase has PhaC and PhaE subunits, whereas group IV PhaC and PhaR. The class III and class IV of PHA synthases were observed in *Allochromatium vinosum* and *Bacillus megaterium*, respectively [41]. The synthesis of polyhydroxyalkanoates by bacteria under laboratory conditions enforces maintaining appropriate conditions such as the proper concentration of carbon to nitrogen, the presence of macro and microelements, the optimum temperature and pH, the proper concentration of inoculum [42]. Moreover, researchers reported that unbalanced growth conditions are more favorable to efficient PHAs synthesis and accumulation [43,44]. However, a bacterial species produced the PHAs in the biotechnological processes is the most important factor. Many microorganisms have been studied for PHA synthesis, but only a few of them have a promising ability to produce these biopolyesters. Many bacteria did not meet the crucial criteria, such as the capability to metabolize raw carbon sources to achieve a satisfied growth rate and a high PHAs productivity (Figure 1).

Furthermore, to compete with synthetic plastics the overall PHAs production costs should be decreased by using inexpensive feedstocks. Therefore, many scientific groups have focused their attention on the possibility of using agro-industrial waste streams for fermentation processes to make PHAs economically feasible [45]. A number of bacterial species have been reported to synthesize PHAs in satisfactory amounts utilizing waste from the various agrofood industry (Table 3).

## 3. PHAs Biosynthesis Using By-Products of the Dairy Industry

The global production of by-products from the dairy industry reached 180–190 million tons per year [83]. During cheese production, whey as a byproduct is generated in high volumes. It is a waste liquid derived from cheese manufacturing that seems to be an appropriate substrate for PHAs synthesis. It contains mainly lactose (44–46%), proteins (6–8%), and lesser amounts of lactic acid (0.5–6.4%), fats (0.06–0.8%), riboflavin (vitamin B2), mineral salts such as NaCl and KCl (0.46–10%), calcium (1.2–1.6%), and phosphate (2.0–4.5%) [46]. There are a number of publications that proved the possibility of using cheese whey (CW) as a carbon source for the growth and PHAs synthesis and accumulation by bacteria. The value of using CW and its derivatives is known because it would decrease the overall costs of PHA production and solve an environmental problem caused by their high organic load. 

However, finding bacteria able to produce PHAs when growing on whey is challenging due to the fact that some microorganisms regarded as the best PHA producers were uncapable of directly metabolizing whey towards PHA synthesis. It is known that *Cupriavidus necator* or *Alcaligenes eutrophus* are able to accumulate up to 80% of PHAs cultured on glucose, but they are unable to grow and synthesize these biopolyesters using lactose, the predominant carbon source of whey [84]. It was proved that only Alcaligenes latus known as the efficient PHA producer was capable of converting whey lactose into P(3HB) homopolymer resulting in its final concentration of 1.28 g L^−1^ and the productivity level of 0.11 g L^−1^ h^−1^ [85]. Many studies reported that due to complexity of whey, there was a need to conduct its series of pretreatments. Das et al. [46] revealed that ultrafiltered cheese whey better supported the P(3HB) synthesis and accumulation than the whole cheese whey. The authors confirmed that *Bacillus megaterium* NCIM 5472 was able to accumulate up to 75.5% of P(3HB) homopolymer of its cell dry weight generating a P(3HB) yield of 8.29 g L^−1^. It is so far the highest reported P(3HB) concentration. The above-mentioned results proved that after ultrafiltration some proteins that reduced the inherent nitrogen concentration in media could be removed and in consequence an increase in the homopolymer concentration could be observed. Furthermore, the attempts were made to reduce the proteins content by acidifying whey to a pH close to 4 [50]. After a heat treatment, centrifugation and filtration, the resulted whey supernatant was used as a carbon source in a culture medium. However, it was observed that the removal of most whey proteins led to a decrease in the growth of *Methylobacterium* sp. ZP24 lowering the final polymer yield [86]. Furthermore, *Thermus thermofilus* HB8 efficiently converted whey supernatant into a PHA heteropolymer containing the short chain length 3-hydroxyvalerate (3HV; 38 mol%) and the medium chain length, 3-hydroxyheptanoate (3HHp; 9.89 mol%), 3-hydroxynanoate (3HN; 16.59 mol%), and 3-hydroxyundecanoate (3HU; 35.42 mol%) [50]. After 24 h of cultivation, the authors determined 35.6% of this copolymer with unique composition [50]. Furthermore, promising results were published by Obruca et al. [47] who improved P(3HB) yields about 50-fold in *Bacillus megaterium* CCM 2037 grown on cheese whey supernatant by supplementing the culture medium with 1% of ethanol at the beginning of the stationary phase of the bacterial growth. Recently, NCIM 5472 strain of *Bacillus megaterium* was tested towards scl-copolymer production using cheese whey permeate and propionic acid as a co-substrate. It was confirmed that the highest P(3HB-co-3HV) content of 86.2% of cell dry weight (CDW), and its yield of 3.0 g L^−1^ was achieved in the cultivation supplemented with 0.1% of propionic acid [52].

Moreover, to improve the PHAs productivity by highly PHA-producing bacteria that are not capable of direct lactose utilization, the chemical or enzymatical conversion of whey lactose into glucose and galactose prior to cultivation processes was conducted. Kucer et al. [48] proved that *Halomonas halophila* was capable of metabolizing whey lactose after the HCl-catalyzed hydrolysis and producing P(3HB) at the concentration of 3.26 g L^−1^. Moreoever, Pais et al. [53] described that chemically hydrolyzed cheese whey could be efficiently metabolized by *Haloferax mediterranei* towards P(3HB-co-3HV) production. The conducted batch cultivation confirmed that this bacterium reached an active biomass concentration of 7.54 g L^−1^ with a copolymer content of 53% of CDW, and a volumetric productivity of 4.04 g L^−1^ day^−1^. Some authors succeeded in the PHA production using glucose and galactose originated from enzymatic hydrolysis of cheese whey lactose. *Haloferax mediterranei* DSM 1411 was reported to be capable to accumulate up to 66% of P(3HB-co-3HV) copolymer [87]. Nevertheless, it should be highlighted that the use of hydrolysis (especially enzymatic hydrolysis) greatly increases the overall costs of a PHA production process. Therefore, there is a need to look for microorganisms that accumulate high levels of these biopolyesters using whole cheese whey as a carbon source. Bustamante et al. [49] revealed that *Caulobacter segnis* DSM 29236 in the fed-batch fermentation reached up to 9.25 g L^−1^ of P(3HB) by directly hydrolyzing lactose from whey. So far, it is the highest reported P(3HB) concentration in the growth of pure bacterial culture using whole cheese whey without any pre-treatments processes. Lower content of PHAs (14% of CDW) was synthesized in *Pseudomonas aeruginosa* MTCC 7925A cells grown on cheese whey. However, in this case, a unique SCL–LCL-PHA co-polymer was produced consisting of 3-hydroxybutyrate (79.2 mol%), 3-hydroxyvalerate (10.5 mol%) and lesser amounts of 3-hydroxyhexadecanoate (1.9 mol%) and 3-hydroxyoctadecanoate (8.4 mol%) [54]. The above-mentioned results suggested that only a few wild-type bacteria are capable of producing PHAs from whey without the costly series of pre-treatments; however, the PHAs content in their cells was reported to be low. Therefore, it is essential to conduct an extensive bioprospecting of microbes from nature to find bioproducers that will be able to directly metabolize whey lactose and generate high PHA yields. 

## 4. Biodiesel Derived Glycerol as a Feedstock for PHA Production

Waste glycerol from biodiesel is generated in a large quantity as a major byproduct from biodiesel manufacturing and can be used in the pharmaceutical or cosmetics industry. However, it is not economically feasible to refine crude glycerol to high purity for commercial applications [88]. Furthermore, disposal of crude glycerol is associated with a fee and creates environmental concerns [89]. Therefore, many studies have been focused on the finding a way to utilize low-value waste glycerol and produce value-added bioproducts. It has been successfully adopted in green technologies for conversion into PHAs (Table 3). The microbial potential to produce PHAs using waste glycerol derived from biodiesel industry was comprehensively reviewed by Zhu et al. [90] who described the research results obtained from the fermentations processes to year 2013. Therefore, below we provide an overview of trends towards PHAs synthesis using waste glycerol based on selected papers from numerous scientific journals published after year 2013. It was demonstrated that P(3HB) homopolymers, the most common polyhydroxyalkanoate in nature, could be efficiently produced by marine bacteria grown on biodiesel-derived glycerol. Promising results have been published by Takahashi et al. [28] who demonstrated that two isolates LAMA 677 and LAMA 685 from the Mid-South Atlantic ridge cultivated on a medium supplemented with residual glycerol were able to accumulate in their cells up to 52.9 and 53.6% of P(3HB), respectively. It was revealed that nutrients limitation stimulated the synthesis of this homopolymer. In the cultivation of *Aeromonas* sp. AC_03, higher homopolymer content in the bacterial cells was observed under a phosphorus-limiting environment (22% of CDW) compared to nitrogen starvation (5.6% of CDW) [91]. Moreover, the PL26 strain belonging to *Bacillus licheniformis* species was found to be an efficient (P3HB-co-3HV) producer and was capable of synthesizing and accumulating up to 64.6% [92]. Higher content of this copolymer was reported by Kumar et al. [93] who revealed that *Bacillus thuringiensis* EGU45 produced up to 74.8% of (P3HB-co-3HV) after 72 h of the cultivation. Furthermore, the authors observed that PHAs were accumulated in the quite consistent content in the bacterial cells over a period of 96 h suggesting that this bacterium could tolerate high levels of crude glycerol that supports efficient copolymer production. It is especially important regarding large scale production, where the flexibility of harvesting time without affecting the biopolymers, productivity can be considered as great assets. Moreoever, the glycerol-rich-phase from a biodiesel plant supported the growth and the efficient synthesis of P(3HB-4HB-3HV) terpolymers by *Cupriavidus necator* DSM 545 [94]. In addition, PHA_MCL_ with a high percentage of unsaturated monomers were synthesized by *Enterobacter* sp. ASC3 isolated from soil that was contaminated with spent cooking oil [62]. This bacterium was capable of accumulating 17.5 g L^−1^ of P(3HO-co-3H5DD) copolyesters. Higher 3H5DD content (>99 mol%) was reported in the PHA_MCL_ extracted from *Acinetobacter* sp. ASC1 and *Bacillus* sp. ASC4. The unsaturated side chain of 3H5DD monomers improved the properties of the extracted PHA_MCL_ and extended their potential future applications. It was also proved that *Cupriavidus necator* IPT027 synthesized up to 71.1% of copolymers containing novel PHA-constituents as building blocks of medium chains [3HTD (5.54–13.10 mol%)] and long chains [11HHD (53.89–69.05 mol%), 15HPD (4.29–6.04 mol%)] [58]. The authors observed that the composition of crude glycerol influenced not only the content of the monomers in the extracted biopolymers but also their thermal properties. 

It should be highlighted that the microbial growth rate of microorganisms grown on biodiesel-derived glycerol could be affected by different impurities such as alcohol, organic and inorganic salts, heavy metals, polyol or ash. It was reported that the high concentration of NaCl in crude glycerol could inhibit bacterial growth and also have a significant effect on PHA productivity and the yield of a final bioproduct [95].

## 5. Molasses as a Carbon Source for PHA Production

Molasses emerges as an efficient carbon source for PHA synthesis and accumulation because of its nutritional composition, rich in sugar, protein, amino acids and microelements. Given the large market of sugar manufacture, it is possible to obtain residual molasses, from the sugar refinement process, at a low cost. Promising results were published by Kulpreecha et al. [64] who demonstrated that the PHA productivity during *Bacillus megaterium* BA-019 cultivations depended on the type of the conducted cultures. The authors revealed that fed-batch cultures with molasses enhanced cell dry mass concentration and P(3HB) productivity up to 72.6 g L^−1^ and 1.27 g L^−1^ h^−1^, respectively. The same observations were made by Desouky et al. [63] who noticed that also *Bacillus flexus* AZU-A2 produced higher level of P(3HB) (6.13 g L^−1^) with its recovery yield of 92.1% of CDW in fed-batch fermentation compared to batch strategy. Furthermore, the ability to synthesize and accumulate P(3HB) homopolymer during the utilization of sugarcane molasses was found in other species, however in much lower concentrations: *Cupriavidus necator* [66,96] *Bacillus subtilis* BPP-19 and BPPI-14 [69]. 

Besides homopolymers, some microorganisms were found to be able to synthesize scl-mcl-PHA copolymers using molasses as the only carbon source. Hassan et al. [70] observed that *Clostridium beijerinckii* ASU10 (KF372577) has the potential to produce P(3HB-co-3HO) in the amount of 37.93% of CDW. Gas chromatography–mass spectrometry (GC-MS) analysis confirmed that the extracted copolymer consisted of 47.3 mol% of 3HB and 52.7% of 3HO. 

The above-mentioned results seem to be promising; however, some types of molasses such as soy molasses, a byproduct of the soybean processing industry, could be not only a rich source of growth promoters but also could contain high concentrations of growth inhibitors. Therefore, as a substrate in bacterial cultures must be diluted several times to reduce the inhibitory effects prior fermentations processes where it is used as a nutrients source [97]. 

## 6. Spent Oils as Substrate for PHA Biosynthesis

Various waste oils have been proven to be the effective carbon sources towards PHA biosynthesis. These feedstocks are considered as the alternative carbon sources for the production of bacterial biopolyesters due to their low market prices, continuous availability and global price consistency. The main bottleneck of using spent oils towards PHA production is their possible inconsistency and heterogeneity due to the exposure to high temperatures, food and air for long periods. Furthermore, chemical reactions during a frying process can form toxic compounds that could hamper the growth of PHA producers. Therefore, the evaluation of characteristics and fatty acid composition of frying oils seems to be essential to optimize the PHAs synthesis process.

*Cupriavidus necator* is usually employed for PHA production on oily substrates because it was observed that this bacterium was able to utilize triacylglycerols efficiently and was used as a lipid-based PHA producer synthesizing high product yields [98]. The highest P(3HB) content of 89.1% with CDW of 55.4 g L^−1^ was determined in the fed-batch culture of *Cupriavidus necator* H16 grown on spent coffee grounds oil [76]. The authors proved that in comparison to other types of waste oils, the utilization of coffee oil resulted in the highest biomass as well as P(3HB) productivity. Recently, PHA accumulation under high salinity seems to be a trendy topic. Halophiles are considered as the “coming stars” for industrial biotechnology because as the extremophilic strains provide many benefits such as the resistance to microbial contamination, the reduction of sterility demands the possibility of a continuous mode of cultivation [99]. There are many reports suggesting that halophiles are capable of converting various inexpensive carbon sources into value-added PHAs; however, a few of them proved that these bacteria valorized efficiently lipid substrates. Kumar and Kim [75] confirmed that *Paracoccus* sp. LL1 has the potential to serve as a single cell factory for bioconversion of waste cooking oil into high value PHAs. The strain produced 1 g L^−1^ of P(3HB-co-3HV) corresponding to 30.9% of CDW. Recently, for the first time, different *Halomonas* sp. were investigated towards their potential to be employed for efficient biotechnological conversion of waste frying oil into polyhydroxyalkanoates [73]. *Halomonas neptunia* and *Halomonas hydrothermalis* were identified as good PHA producers with the potential to synthesize about 23% of P(3HB). Moreover, the study showed that initial concentration of NaCl was crucial for PHA yields as well as molecular weight of the extracted polymers. The decrease in NaCl concentration not only improved the biopolyester productivity but also resulted in substantial increase in average molecular weight (Mw) of the produced polymer (from 245 kDa at 100 g L^−1^ NaCl to 381 kDa at 40 g L^−1^ NaCl), which is beneficial taking into account thermal processing of this material, where high molecular weight is desired. Furthermore, *Halomonas hydrothermalis* was reported to biosynthesize P(3HB-co-3HV) (68.83% of CDW), when valerate was utilized as a precursor, with the 3HV fraction in the copolymer reached high values of 50.15 mol% [81]. Van Thuoc et al. [31] found that *Salinivibrio* sp. M318, a moderately halophilic bacterium isolated from fermenting shrimp paste, was able to use a mixture of waste fish oil and glycerol towards PHA synthesis. The results proved that this strain synthesized up to 51.5% of CDW in the fed-batch culture after 78 h of the fermentation proving that it is a promising wild-type bacterium for the production of PHA from aquaculture residues. Moreover, the highest biosynthesis of poly(3-hydroxybutyrate-co-4-hydroxybutyrate) [P(3HB-co-4HB)] (54.6% of CDW) was reached when 1,4-butanediol was added as a precursor to the culture medium. The comparable content of P(3HB-co-3HV) copolymer (52.4% of CDW) was determined when sodium valerate was supplied. To the best of our knowledge, the highest content of a scl-copolymer was observed in the cultivation of *Bacillus thermoamylovorans* supplemented with waste cooking oil. Sangkharak et al. [74] revealed that this bacterium was able to produce up to 87.5% of P(3HB-co-3HV)] with 85 mol% of 3HB and 15 mol% of 3HV content. Moreover, the authors proved that the extracted copolymer can be used as a substrate for P(3HB-co-3HV) methyl ester (3-HAME) production, a biofuel. It has been demonstrated that the properties of 3HAME pass ASTM and Thailand’s fuel standards. 

Especially, bacteria belonging to Pseudomonas sp. were reported to be capable of utilizing inexpensive oils into PHA_MCL_. Ruiz et al. [100] conducted pulse fed-batch fermentation using *Pseudomonas chlororaphis* 555 grown on waste cooking oil. The authors achieved the highest reported biomass (73 g L^−1^) and PHA volumetric productivity (0.29 g mcl-PHA^−1^L^−1^ h^−1^). Furthermore, they detected 3-hydroxyoctanoic acid and 3-hydroxydecanoic acid monomers followed by 3-hydroxydodecanoic acid monomers as the dominant subunits with lesser amounts of 3-hydroxyhexanoic acid in the synthesized biopolymer. In addition, saponified waste palm oil seemed to be also a suitable substrate for PHA_MCL_ biosynthesis applying fed-batch fermentations of *Pseudomonas* sp. Gl01. This strain was able to synthesize up to 48% of PHA_MCL_ at the early stage of the culture (at 17 h) with the monomeric composition contained 3-HHx, 3-HDD, and 3-HTD. Furthermore, due to presence of uneven fatty acids in the waste feedstock, the authors reported a high molar fraction of 3HN (up to 63 mol%) what indicated in the waste palm oil used [91].

## 7. Other Agro-Food Industrial Wastes as Feedstock for PHA Production

There are many reports confirming that other wastes from the food industry could replace the costly sugars and pure fatty acids commonly used as substrates in the fermentations towards PHA production. The grape pomace, a by-product of wine production, was reported to be efficiently converted by bacteria into PHAs. The attention was focused on this type of a feedstock due to its high concentration of fermentable sugars (mainly glucose and fructose) and the fact that it could be used directly without additional, costly saccharification step. So far, only a few papers described the use of grape pomace as a carbon source for the biosynthesis of PHAs. Kovalcik et al. [78] compared the potential of *Cupriavidus necator*, *Halomonas halophila* and *Halomonas organivorans* to produce P(3HB) by using oil and fermentable sugars derived from grape pomace. However, *Cupriavidus necator* was found to be the best producer that after 29.5 h of the biofermentation was able to produce 8.5 g L^−1^ of P(3HB) corresponding to 63% of CDW. Moreover, grape pomace sugar extract was converted into PHA_MCL_ by *Pseudomonas* species. Follonier et al. [101] proved that during a two-step fermentation process *Pseudomonas resinovorans* hydrolyzed grape pomace reaching the final biomass concentration of 6.1 g L^−1^ and _PHAMCL_ accumulation at the level of 21.3 g L^−1^. The same authors demonstrated the scalability of the above approach by conducting a two-step fermentation in 100 L bioreactor achieving PHA_MCL_ with a high degree of unsaturation. They achieved 14.2 g L^−1^ of CDW containing 41.1% of poly(3-hydroxyoctanoate-co-3-hydroxy-10-undecenoate) with 53 mol% and 47 mol% of saturated and unsaturated monomers, respectively [102]. 

Moreover, some fruit peels were successfully transformed by microorganisms into valuable polyhydroxyalkanoates. Vijay and Tarika [27] demonstrated that banana peel can be efficiently utilized by *Geobacillus stearothermophillus*. It has been shown that this soil bacterium was able to synthesize up to 84.63% of P(3HB) at 96 h of the cultivation. The same authors found that *Bacillus subtilis* accumulated a comparable content of P(3HB) at 24 h of the fermentation (71.8% of CDW). *Bacillus thuringiensis* was found to be the lesser efficient PHA_SCL_ producer. In the cultivation supplemented with mango peel, it reached a cell dry mass of 7.9 g L^−1^ with 51.3% of P(3HB) [103]. 

The halophilic bacteria effectively utilized different agro-industrial hydrolysates and effluents. Pleissner et al. [104] extracted 2.6 g L^−1^ of P(3HB) from the batch cultivation of *Halomonas boliviensis* supplemented with bakery waste hydrolysate. The same species was able to synthesize the scl-homopolymer in lesser concentration during fed-batch fermentation (2.1 g L^−1^ of P(3HB)). A marine bacteria, *Halomonas* sp. SF2003 seems to be a potential P(3HB) bioproducer in a batch culture fed with agro-industrial effluent. Lemechko et al. [29] found that this halophile converted the effluent from fruit industry into P(3HB) achieving the concentration rate of 1.3 g L^−1^ at 40 h of the culture. Furthermore, the addition of valeric acid enabled the authors to obtain P(3HB-co-3HV) copolymer with controlled proportion of the 3HV monomer. Recently, it was also revealed that rice mill effluent could be efficiently fermented by *Acinetobacter junii* to P(3HB) in the concentration of 2.4 g L^−1^ corresponding to 85.7% content of this homopolymer in the bacterial cells [105]. 

The production of canned pineapples, especially in Asia, reached 28.4 million tons per year generating a vast volume of waste during processing [71]. Therefore, also such wastes were tested towards value-added bioproducts synthesis. Sukruansuwan and Napathorn [79] demonstrated that a maximum biopolymer content of 60% of CDW was reached by *Cupriavidus necator* when grown on crude aqueous extract originated from canned pineapple production. The authors revealed that nitrogen-limiting conditions influenced positively on PHA concentration. A similar observation was made by Amini et al. [106] who demonstrated the same bacterium produced P(3HB) in higher concentration cultured on beer brewery wastewater under nutrients limitation. Moreover, nitrogen limitation supported the scl-homopolymer synthesis more efficiently in the culture of *Burkholderia sacchari* on waste paper hydrolase [107]. 

Coffee is one of the most valuable products worldwide. Dynamic industrialization and population growth contribute to increase coffee production. However, this manufacture generates a lot of coffee wastes and byproducts among which some of them cannot be reused. Only a few studies presented the possibility of the utilization of coffee wastes to biopolymers production. Obruca et al. [81] proved that spent coffee grounds are an efficient substrate for the bacterial growth and P(3HB) synthesis. The authors demonstrated that *Cupriavidus necator* was able to accumulate up to 89.1% of PHA_SCL_ in a fed-batch cultivation. The final biomass concentration reached 55.4 g L^−1^ corresponding to PHAs productivity of 1.33 g L^−1^ h^−1^. Moreover, Kovalcika et al. [80] studied the potential of *Halomonas halophila* to produce PHAs from fermentable sugars derived from spent coffee grounds (SCGs). This organism was able to process SCG hydrolysates as a carbon source for its growth, however to prevent the growth inhibition, the hydrolysates have been detoxified using sorbent based on styrene-divinylbenzene based resins. The authors successfully reached 0.95 g L^−1^ of P(3HB) with its content in bacterial cells of 27% of CDW and the molecular weight of about 440–825 kDa. 

## 8. PHA Production by Genetically Modified Bacteria Using Agro-Industrial Wastes

Many efforts have been made to improve PHA biosynthesis by using genetically recombinant strains (Table 4). Due to ease of molecular manipulations, *Escherichia coli* is becoming the most promising host bacterium for PHAs production. *Escherichia coli* mutants result in the best PHA production with whey as a carbon source. Furthermore, hydrolysis steps are not necessary because E. coli possesses enzymes for the breakdown of lactose [108]. According to Pais et al. [109] a recombinant *Escherichia coli* CML3-1 fused to a lactose-inducible promoter, into the chromosome, via transposition mediated mechanism and harboring PHA synthesis genes from *Cupriavidus necator* was able to produce 7.83 g L^−1^ of P(3HB) cultured on cheese whey. Moreover, the influence of molasses on the growth and PHA synthesis by recombinant *Escherichia coli* harboring *phaC1* gene of *Pseudomonas* sp. LDC-5 was investigated. The PHA yield was higher using a substrate derived from sugar refinery mill (75.5% of CDW) as a carbon source compared to sucrose (65.1% of CDW) [110]. Moreover, biodiesel derived glycerol was successfully employed towards the production of poly(3-hydroxybutyrate)-block-poly(3-hydroxypropionate) copolymer (P3HB-b-P3HP) with varied composition by *Escherichia coli* HBP01 contained PHA synthase gene of *Cupriavidus necator* and two parallel synthetic pathways to produce the 3HB and 3HP monomers, respectively.

It was confirmed that *Cupriavidus necator* could also be an appropriate host for the PHA synthesis. *Cupriavidus necator* Re2058/pCB113 harboring PHA synthase gene of *Rhodococcus aetherivorans* I24 and an enoyl coenzyme A (enoyl-CoA) hydratase gene (*phaJ*) from *Pseudomonas aeruginosa* was able to facilitate synthesis of P(3HB-co-3HHx) grown on waste frying oil and low quality waste animal fats and tallow. The mutant synthesized up to 60% of the scl-mcl-copolymer with a final HHx concentration of 19 mol%. However, the HHx content decreased continuously over the cultivation time [116]. The same mutant produced up to 74% of P (78 mL% 3HB-co-22 mol% 3HHx) grown on sludge palm oil, a difficult-to-be-used solid byproduct of the palm oil milling industry [116]. The other recombinant *Cupriavidus necator* PHB¯4 harboring the PHA synthase gene of *Aeromonas caviae* (PHB¯4/pBBREE32d13) reached in its cells up to 85% of P(3HB-co-3HHx) copolymers cultured on waste cooking oil as the only carbon source [114]. Whey was also successfully employed for PHAs synthesis by recombinant *Cupriavidus necator*. Povolo et al. [84] has proved that *Cupriavidus necator* DSM 545 was able to produce P(3HB) after genetic modification made by an insertion of the lac operon within depolymerase gene (*phaZ*). When using whey permeate the profiles of growth, and polyester synthesis and accumulation were higher (22% of CDW after 48 h growth) than those obtained in lactose as a control. 

Obruca et al. [111] engineered *Cupriavidus necator* H16 by random chemical mutagenesis that significantly enhanced specific activities of enzymes engaged in oxidative stress response. The recombinants were capable of producing P(3HB) more efficiently compared to the wild-type strain reaching 7.6 g L^−1^ and 4.9 g L^−1^, respectively. Moreover, UV mutations have been proven to be useful for enhanced PHAs production. De Paula et al. [119] using UV radiation obtained *Pandoraea* sp. mutants being able to produce up to 53% of P(3HB-co-3HV) copolymer using crude glycerol from biodiesel industry. Furthermore, sequential mutation of *Bacillus licheniformis* PHAs-007 using UV and N-methyl-N’-nitro-N-nitrosoguanidine (NTG) improved the level of the synthesis and accumulation of the resulted PHA copolymer. The *Bacillus licheniformis* M2-12 transformant was able to produce about 2.94 times more copolymers in comparison to the PHAs-007 parent strain. The PHA concentration increased up to 16.23 g L^−1^ when the culture was supplemented with palm oil mill effluent (POME) as the sole carbon source. Furthermore, gas chromatography analysis confirmed that M2-12 mutant produced a P(3HB-co-3HV-co-3HH) copolymer grown on POME whereas the wild type synthesized a copolymer consisting of 3HB and 3HV monomers [74]. 

Moreover, engineered metabolic pathways have proven to enhance PHA biosynthesis using waste. Borrero-de Acuña et al. [117] knocked out the *tctA* gene in *Pseudomonas putida* KT2440, encoding for an enzyme of the tripartite carboxylate transport system, to improve PHA_MCL_ synthesis. The authors observed a nearly two-fold increment in the PHA volumetric productivity. The recombinant *P. putida *Δ*tctA* strain synthesized up to 1.91 g L^−1^ after 72 h cultivation on 20 g L^−1^ of waste vegetable oil whereas the wild type produced only 1.01 g L^−1^ of PHAs. Moreover, it was demonstrated that the disruption of the gene responsible for the depolymerization process of PHA (PHA depolymerase, *phaZ*) in *Pseudomonas putida* KT2440 improved the biopolymer titer to the level of 47% of CDW when cultured on biodiesel-derived glycerol [59]. In addition, Sharma et al. [118] proved that a phenazine-deficient mutant of *Pseudomonas chlororaphis* PA23-63 was able to synthesize PHA_MCL_ at the higher level of 24.8% of CDW compared to the wild-type (10.5% of mcl-PHAs) with a biomass concentration of 4.1 g L^−1^ when grown on waste frying oil. The extracted PHA_MCL_ composition consisted of a mixture of 3HHx, 3HO, 3HD, 3HDD and 3HTD.

## 9. Challenges and Future Prospects in PHA Production Using Waste Streams

**Figure 2:**
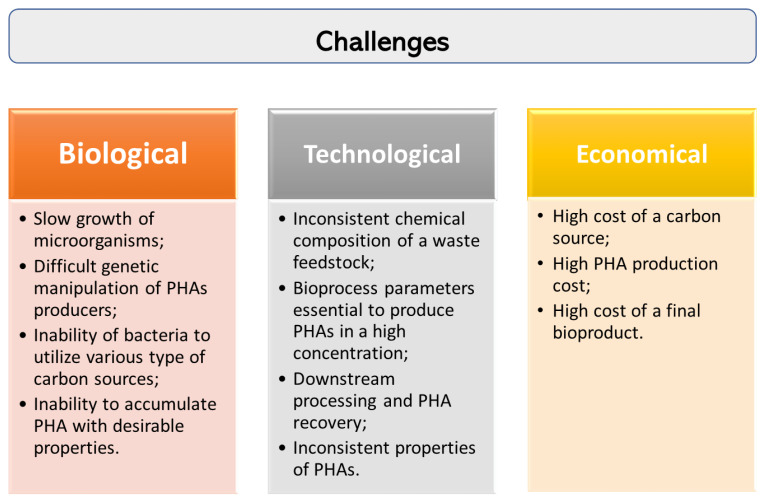
Challenges in PHA production from waste carbon sources.

One of the major issue in the commercialization of PHAs is their overall production cost. During the past decades, great progress has been made in PHA-related research regarding its biosynthesis and possibility to convert different industrial wastes into the value-added biopolymers. However, there are still a number of challenges that need to be addressed to achieve efficient production of PHAs from wastes as feedstocks (Figure 2).

Generally, wastes generated from different industrial companies could have a different chemical composition or some impurities and in consequence can be converted in PHAs with varied efficiency, structure and properties. It is a huge challenge taking into account a large scale process of PHA production. Furthermore, bacteria are not able to metabolize wastes in high amounts. Therefore, research is still needed to be done to adjust a suitable pretreatment method of waste feedstock and to optimize operational parameters and nutrients composition during fermentation processes. Moreover, the development of fermentation strategies and the implementation of genetic engineering tools are essential to enhance PHA productivity.

Moreover, only a few bacteria, able to synthesize PHAs, can be considered useful for biotechnological production of these biopolymers on a commercial scale. There is still a growing need to find new bacteria possessing the potential to produce PHAs when grown on industrial waste. They should be evaluated concerning growth rate on waste fluxes, the ability to wastes conversion into PHAs, ease for genetic manipulation or risk of contamination during bioprocesses. Furthermore, it is crucial to understand their metabolic cycles to utilize waste for potential industrial use due to their high stability under unfavourable growth conditions. 

By applying metabolic engineering strategies, the PHA efficiency was successfully improved, and it was also demonstrated that the monomer composition of the produced PHAs can be tuned [59]. However, there is a shortage of studies that aim at using recombinants to produce PHAs at a large scale. Some bacterial species like *Escherichia coli* or *Cupriavidus necator* seem to be the potential cell factories for PHA synthesis and accumulation. They can be used as the industrial producers due to their available genomic information, ease for genetic manipulation, high PHA content of cell dry weight, fast growth, and high substrate utilization towards PHAs. Moreover, genetic recombination of biosynthetic pathways could enhance the final biopolymers productivity. Progress in molecular biology helped to improve the ability of bacteria to produce PHA at a high concentration. However, a deeper understanding of genetic and metabolic regulation that drive this biopolyesters synthesis is essential to construct a bacterial platform that will be capable of producing defined PHAs structures and predictable material properties which determine their further applications. More data are needed to be generated from studies with a multi-omics-focused design, to provide more understanding of metabolic pathways response to waste feedstocks used in the microbial PHA production processes. Moreover, synthetic biology tools may be applied to accelerate the bacterial cell growth by changing the binary fission to multiple fission achieving cell division into more than two daughter cells at same time and improved PHA production rate [118]. 

The PHA extraction process from bacterial cells is also a challenging task. It was proved that the price of the PHA separation could reach 30% of the overall biosynthesis costs [119]. In downstream processing, the extraction step using solvents or detergents is still the most widely used for the recovery of PHAs after the fermentation process. First of all, this strategy is expensive considering an industrial scale and harmful to the environment due to additional waste created after biopolymers recovery step. Therefore, green and environment-friendly extraction has been proposed in multiple recent studies as the solution for toxicity of the chemicals employed, their ecological impact and sustainability. The recent developments of ecofriendly solvent extraction and purification processes were comprehensively reviewed by Li and Wilkins [120]. Furthermore, a new autolytic cell disruption system was proposed as a possible method to control and facilitate the release of PHA granules to the extracellular medium. This approach was successfully tested in programmed self-disruptive *Pseudomonas putida* BXHL strain, derived from *P. putida* KT2440, which exhibit alterations in outer membrane integrity that induce lysis hypersensitivity [121]. Moreover, the PHA content in bacteria and its molecular weights can be improved by CRISPRi system. This technology opened a new field in the manipulation of the PHA synthesis-related genes [122]. 

Utilization of different waste streams as feedstocks in the bacterial fermentations towards PHA production will not only reduce the bioprocess costs but also meet the criterium of wastes cycle which is one of the vision of circular economy.

## Figures and Tables

**Table 1:** Manufacturers producing PHAs from renewable resources.

Manufacturer	Carbon Source	Year	References
Bio-on (Italy)	renewable sources or agricultural waste	2007	[8]
Hydal (Chech)	waste cooking oil	2012	[9]
Full-Cycle Bioplastic (USA)	food waste	2015	[10]
Biocycle (Brazil)	sugar cane	2000	[11]
Danimer Scientific ( USA)	canola oil	2007	[12]
Tianjin Green Bioscience (China)	plants	2013	[13]
Tianan Biologic Material Co., (China)	dextrose derived from corn or cassava delivered from China	2004	[14]

**Table 2:** Possible applications of various types of PHAs.

Type of PHAs	Application	References
PHA_SCL_		
Poly(3-hydroxybutyrate)	Blood components, tissue engineering, materials for medical devices, drug delivery system, food packaging	[19,32,33,34]
Poly(4-hydroxybutyrate)	Agricultural nets	[35]
Scl-copolymers		
Poly(3-hydroxybutyrate-4-hydroxybutyrate)	Matrices, microspheres or micro capsules	[36]
Poly(3-hydroxybutyrate-co-3-hydroxyvalerate)	Drug carriers, e.g., in cure of chronic and implant osteomyelitis	[37]
Poly(3-hydroxybutyrate-cco-3-hydroxyvalerate) with the addition of poly (L-lactic-co-glycolic acid)	Microspheres applied in medicine and tissue engineering	[38]
Scl-mcl-copolymers		
Poly(3-hydroxybutyrate-co-3-hydroxyhexanoate)	Scaffolds applied in medicine and tissue engineering	[39]
Poly(3-hydroxyoctanoate)/poly(3-hydroxybutyrate) blends	Nerve tissue engineering	[40]

**Table 3:** Comparison of PHAs production processes by pure bacteria cultures from waste substrate.

Bacteria	Carbon Source	Cultivation Mode	Biomass (g L^−1^)	PHA Content (%)	Type of PHAs	References
**Dairy industry wastes**						
*Bacillus megaterium* NCIM 5472	Cheese whey permeate	Batch	11.0	75.5	P(3HB)	[46]
*Bacillus megaterium* CCM 2037	Whey supernatant	Batch	2.9	51.0	P(3HB)	[47]
*Halomonas hydrophila*	Cheese whey chemically hydrolysated	Batch	8.5	38.3	P(3HB)	[48]
*Caulobacter segnis* DSM 29236	Cheese whey	Fed-batch	25.0	37.0	P(3HB)	[49]
*Thermus thermophilus* HB8	Whey supernatant	Batch	1.6	35.6	P(3HB)	[50]
*Burkholderia sacchari* LMF 101	Cheese whey + glucose	Batch	1.5	5.1	P(3HB)	[51]
*Bacillus megaterium*	Cheese whey	Batch	3.6	86.6	P(3HB-co-3HV)	[52]
*Haloferax mediterranei*	Cheese whey	Batch	7.5	53.0	P(3HB-co-3HV)	[53]
*Pseudomonas aeruginosa MTCC*	Cheese whey	Batch	0.2	13.2	P(3HB-co-3HV-co-3HHD-co-3HOD)	[54]
**Biodiesel industry derived byproducts**						
*Marine strain* LAMA 685	Waste glycerol	Batch	0.3	53.6	P(3HB)	[28]
*Pandoraea* sp. MA03	Waste glycerol	Batch	4.3	49.0	P(3HB)	[55]
*Burkholderia glumare* MA13	Waste glycerol	Batch	3.9	41.4	P(3HB)	[56]
*Cupriavidus necator*	Waste glycerol from biodiesel industry	Batch	6.8	72.0	P(3HB-co-3HV)	[57]
*Cupriavidus necator* IPT 027	Crude glycerol	Batch	2.4	71.1	P(3HB-co-3HTP-co-15HPD-co-11HHD)	[58]
*Burkholderia cepacia* IPT 438			1.9	67.4	P(3HB-co-3HTD-co-11HHD)	
*Pseudomonas putida* KT2440	Waste glycerol	Batch	4.2	34.5	P(3HTD-co-3HO-co-3HD-co-3H5DD-co-3HDD)	[59]
*Pseudomonas putida* NRRL B-14875	Waste glycerol	Fed-batch	9.0	19.5	P(3HHx-co-3HO-co-3HD-co-3HDD)	[60]
*Pseudomonas chlororaphis* subsp. *aurantiaca*	Waste glycerol	Batch	6.7	17.1	P(3HHx-co-3HTD-co-3HO-3HD-co-3HDD)	[61]
*Enterobacter* sp. ASC3	Waste glycerol	Batch	33.1	47.2	P(3HO-co-3H5DD)	[62]
*Bacillus* sp. ASC4			7.8	34.4		
*Pseudomonas* sp. ASC2			10.7	28.2		
*Acinetobacter* sp. ASC1			8.5	25.4		
**Molasses**						
*Bacillus flexus* AZU-A2	Sugarcane molasses	Batch	4.2	85.1	P(3HB)	[63]
	Sugarcane molasses	Fed-batch	7.1	84.3		
	Sugarcane molasses + acetic acid	Batch	3.8	42.4		
*Bacillus megaterium* BA-019	Cane molasses	Batch	8.8	61.6	P(3HB)	[64]
		Fed-batch	72.6	42.1		
*Bacillus endophyticus*	Sugarcane molasses	Batch	3.7	59.5	P(3HB)	[65]
*Cupriavidus necator*	Vinasse and sugarcane molasses	Batch	24.0	50.0	P(3HB)	[66]
*Bacillus* sp. BPPI-19	Raw sugarcane molasses	Batch	0.4	45.9	P(3HB)	[67]
	Pre-treated sugarcane molasses		4.5	44.7		
*Bacillus subtilis* RS1	Sugarcane molasses	Batch	2.4	41.7	P(3HB)	[68]
*Bacillus subtilis*	Sugarcane molasses	Batch	1.5	16.7	P(3HB)	[69]
*Clostridium beijerinckii* ASU10	Sugarcane molasses	Batch	0.9	37.9	P(3HB-co-3HO)	[70]
**Waste oils**						
*Burkholderia thailandensis* E264	Digestate of chicken manure combined with waste sunflower oil	Batch	12.6	60.0	P(3HB)	[71]
*Ralstonia* sp. M91	Crude fish oil	Batch	5.3	52.0	P(3HB)	[72]
*Halomonas neptunia*	Waste frying oil	Batch	2.9	23.1	P(3HB)	[73]
*Halomonas halophila*			0.9	0.4		
*Salinivibrio* sp. M318	Waste fish oil + glycerol	Batch	10.0	51.7	P(3HB)	[31]
	waste fish oil + glycerol + 1,4-butanediol	Fed-batch	9.8	54.6	P(3HB-co-4HB)	
	Waste fish oil + glycerol + sodium heptanoate	Fed-batch	11.2	53.0	P(3HB-co-3HV)	
*Bacillus thermoamylovorans*	Waste cooking oil	Batch	4.0	87.5	P(3HB-co-3HV)	[74]
*Paracoccus* sp. LL1	Waste cooking oils	Batch	3.24	30.8	P(3HB-co-3HV)	[75]
**Other agro-food wastes**						
*Cupriavidus necator* H16	Oil from spent coffee grounds	Batch	29.4	90.1	P(3HB)	[76]
		Fed-batch	55.4	89.1		
*Cupriavidus necator* CCGUG 52238	Kitchen waste	Fed-batch	14.4	84.5	P(3HB)	[77]
*Cupriavidus necator*	Grape winery waste	Batch	8.3	76.8	P(3HB)	[78]
*Cupriavidus necator*	Banana peel	Batch	ND	79.7	P(3HB)	[27]
*Bacillus siamensis*			ND	77.6		
*Staphylococcus aureus* JH1			ND	70.0		
*Cupriavidus necator* A-04	Pineapple core hydrolysate	Batch	6.1	35.6	P(3HB)	[79]
	Pineapple peel hydrolysate		5.3	12.7		
*Halomonas* sp. SF2003	Agro-industrial wastewaters	Batch	5.7	33.0	P(3HB)	[29]
*Halomonas halophila*	Fermentable sugars from spent coffee grounds	Batch	3.5	27.0	P(3HB)	[80]
*Burkholderia cepacia*	Hydrolysate of spent coffee grounds	Batch	4.9	54.8	P(3HB-co-3HV)	[81]
*Halogeometricum borinquense* strain E3	Cassava waste	Batch	3.4	44.7	P(3HB-co-3HV)	[30]
*Pseudomonas citronellolis*	Apple pulp waste	Batch	4.0	30.0	P(3HD-co-3HHx-co-3HDD-co-3HTD-co-3HO)	[82]

HB: hydroxybutyrate; HV: hydroxyvalerate; HHx: hydroxyhexanoate; HDD: hydroxydodecanoate; HTD: hydroxytetradecanoate; HPD: hydroxypentadecanoate; HO: hydroxyoctanoate; HHD: hydroxyhexadecanoate; HD: hydroxydecanoate; ND: not determined.

**Table 4:** An overview of PHAs production by genetically modified bacteria grown on waste carbon sources.

Bacteria	Carbon Source	Cultivation Mode	Biomass Concentration (g L^−1^)	PHA Content (%)	Type of PHAs	References
*Cupriavidus necator OE1*	Waste frying oil	Batch	8.6	87.9	P(3HB)	[111]
*Escherichia coli*	Sugar cane molasses	Fed-batch	4.0	75.0	P(3HB)	[110]
*Ralstonia eutropha* NCIMB 11599	Wheat bran hydrolysate	Batch	20.0	62.5	P(3HB)	[112]
*Escherichia coli* P8-X8	Cheese whey	Fed-batch	33.1	38.7	P(3HB)	[109]
*Escherichia coli* CML3-1			28.2	21.7		
*Cupriavidus necator* mRePT	Hydrolyzed whey permeate	Batch	8.0	30.0	P(3HB)	[84]
*C. necator Re2058 (pCB113)*	Waste frying oil and waste animal fat	Batch	3.1	78.1	P(3HB-co-3HHx)	[113]
*Cupriavidus necator* PHB-4	Palm oil-based waste cooking oil	Batch	11.6	73.0	P(3HB-co-3HHx)	[114]
*Ralstonia eutropha* Re2133	Coffee waste oil	Batch	0.9	69.0	P(3HB-co-3HHx)	[115]
*Cupriavidus necator* Re2058/pCB113	Sludge palm oil	Batch	4.5	66.0	P(3HB-co-3HHx)	[116]
	Sludge palm oil + tween 20		4.2	58.0		
*Bacillus licheniformis M2-12*	Palm oil mill effluent	Batch	11.6	87.6	P(3HB-co-3HV)	[74]
*Pseudomonas putida*	Waste vegetable oil	Batch	5.0	38.3	P(3HHx-co-3HO-co-3HD-co-3HDD)	[117]
*Pseudomonas chlororaphis PA23-63*	Biodiesel-derived waste free fatty acids	Batch	4.3	26.1	P(3HHx-co-3HO-co-3HD-co-3HDD-co-3HTD)	[118]
*Pseudomonas chlororaphis PA23-63*	Waste canola frying oil	Batch	4.1	24.8	P(3HHx-co-3HO-co-3HD-co-3HDD-co-3HTD	[118]

HB: hydroxybutyrate; HV: hydroxyvalerate; HHx: hydroxyhexanoate; HDD: hydroxydodecanoate; HTD: hydroxytetradecanoate; HO: hydroxyoctanoate; HD: hydroxydecanoate; ND: not determined.

## Data Availability

Not applicable.

## References

[1] Plastics–the Facts 2017 An Analysis of European Plastics Production, Demand and Waste Data. https://www.plasticseurope.org/application/files/1715/2111/1527/Plastics_the_facts_2017_FINAL_for_website.pdf.

[2] Zhao S., Danley M., Ward J.E., Li D., Mincer T.J. (2017). An approach for extraction, characterization and quantitation of microplastic in natural marine snow using Raman microscopy. Anal. Methods.

[3] Sheldon R.A., Norton M. (2021). Green chemistry and the plastic pollution challenge: Towards a circular economy. Green Chem..

[4] Reddy C.S.K., Ghai R., Kalia V.C. (2003). Polyhydroxyalkanoates: An overview. Bioresour. Technol..

[5] Anderson A.J., Dawes E.A. (1990). Occurrence, metabolism, metabolic role, and industrial uses of bacterial polyhydroxyalkanoates. Microbiol. Rev..

[6] Ranganathan S., Dutta S., Moses J.A., Anandharamakrishnan C. (2020). Utilization of food waste streams for the production of biopolymers. Heliyon.

[7] Amaro T.M., Rosa D., Comi G., Iacumin L. (2019). Prospects for the Use of Whey for Polyhydroxyalkanoate (PHA) Production. Front. Microbiol..

[8] Bio-on Industry http://www.bio-on.it/.

[9] Nafigate Corporation a. s https://www.nafigate.com/en/biotechnology-hydal.

[10] Full Cycle Industry http://fullcyclebioplastics.com/;https://fullcyclebioplastics.com/.

[11] Biocycle Industry http://www.biocycle.com.br.

[12] Danimer Scientific, a Biotechnology Company https://danimerscientific.com/about-us/.

[13] Tianjin GreenBio Materials Co., Ltd. (GreenBio) Company http://www.tjgreenbio.com/.

[14] Tian An Biopolymer Company http://www.tianan-enmat.com/.

[15] Sarkar O., Katakojwalaab R., Mohan S.V. (2021). Low carbon hydrogen production from a waste-based biorefinery system and environmental sustainability assessment. Green Chem..

[16] Voros V., Drioli E., Fonte C., Szekely G. (2019). Process Intensification via Continuous and Simultaneous Isolation of Antioxidants: An Upcycling Approach for Olive Leaf Waste. ACS Sustain. Chem. Eng..

[17] Ciesielski S., Pokoj T., Klimiuk E. (2010). Cultivation-dependent and independent characterization of microbial community producing polyhydroxyalkanoates from raw-glycerol. J. Microbiol. Biotechnol..

[18] Kumar M., Rathour R., Singh R., Sun Y., Pandey A., Gnansounou E., Lin K.Y.A., Tsang D.C.W., Thakur I.S. (2020). Bacterial polyhydroxyalkanoates: Opportunities, challenges, and prospects. J. Clean. Prod..

[19] Tan G.Y.A., Chen C.L., Li L., Ge L., Wang L., Razaad I.M.N., Li Y., Zhao L., Mo Y., Wang J.Y. (2014). Start a research on biopolymer polyhydroxyalkanoate (PHA): A review. Polymers.

[20] Boopathy R. (2000). Factor limiting bioremediation technologies. Bioresour. Technol..

[21] Chen G.Q. (2011). Biofuncionalization of polymers and their application. Adv. Biochem. Eng. Biotechnol..

[22] Meereboer K.W., Misra M., Mohanty A.K. (2020). Review of recent advances in the biodegradability of polyhydroxyalkanoate (PHA) bioplastics and their composites. Green Chem..

[23] Barbosa J.L., Perin G.B., Felisberti M.I. (2021). Plasticization of Poly(3-hydroxybutyrate-co-3-hydroxyvalerate) with an Oligomeric Polyester: Miscibility and Effect of the Microstructure and Plasticizer Distribution on Thermal and Mechanical Properties. ACS Omega.

[24] Cinelli P., Seggiani M., Mallegni N., Gigante V., Lazzeri A. (2019). Processability and Degradability of PHA-Based Composites in Terrestrial Environments. Int. J. Mol. Sci..

[25] Koller M., Salerno A., Braunegg G., Stephan K. (2013). Polyhydroxyalkanoates: Basics, production and applications of microbial biopolyesters. Bio-Based Plastics: Materials and Applications.

[26] Shamala T.R., Vijayendra S.V.N., Joshi G.J. (2012). Agro-industrial residues and starch for growth and co-production of polyhydroxyalkanoate copolymer and α-amylase by *Bacillus* sp. CFR67. Braz. J. Microbiol..

[27] Vijay R., Tarika K. (2018). Banana peel as an inexpensive carbon source for microbial polyhydroxyalkanoate (PHA) production. Int. Res. J. Environ. Sci..

[28] Takahashi R.Y.U., Castilho N.A.S., da Silva M.A.C., Miotto M.C., de Souza Lima A.O. (2017). Prospecting for Marine Bacteria for Polyhydroxyalkanoate Production on Low-Cost Substrates. Bioengineering.

[29] Lemechko P., Fellic M.L., Bruzaud S. (2019). Production of poly(3-hydroxybutyrate-co-3-hydroxyvalerate) using agro-industrial effluents with tunable proportion of 3-hydroxyvalerate monomer units. Int. J. Biol. Macromol..

[30] Salgaonkar B.B., Mani K., Bragança J.M. (2019). Sustainable Bioconversion of Cassava Waste to Poly(3-hydroxybutyrate-co-3-hydroxyvalerate) by *Halogeometricum borinquense* Strain E3. J. Polym. Environ..

[31] Van Thuoc D., My D.N., Loan T.T., Sudesh K. (2020). Utilization of waste fish oil and glycerol as carbon sources for polyhydroxyalkanoate (PHA) production by *Salinivibrio* sp. M318. Int. J. Biol. Macromol..

[32] Reis C.P., Neufeld R.J., Ribeiro A.J., Veiga F. (2006). Nanoencapsulation Methods for preparation of drug-loaded polymeric nanoparticles. Nanomed. Nanotechnol. Biol. Med..

[33] Philip S., Keshavarz T., Roy I. (2007). Polyhydroxyalkanoates: Biodegradable polymers with a range of applications. J. Chem. Technol. Biotechnol..

[34] Manikandan N.A., Pakshirajan K., Pugazhenthi G. (2020). Preparation and characterization of environmentally safe and highly biodegradable microbial polyhydroxybutyrate (PHB) based graphene nanocomposites for potential food packaging applications. Int. J. Biol. Macromol..

[35] Williams S.F., Rizk S., Martin D.P. (2013). Poly-4-hydroxybutyrate (P4HB): A new generation of resorbable medical devices for tissue repair and regeneration. Biomed. Tech..

[36] Nigmatullin R., Thomas P., Lukasiewicz B., Puthussery H., Roy I. (2015). Polyhydroxyalkanoates, a family of natural polymers, and their applications in drug delivery. J. Chem. Technol. Biotechnol..

[37] Yagmurlu M.F., Korkusuz F., Gürsel I., Korkusuz P., Ors U., Hasirci V. (1999). Sulbactam cefoperazone polyhydroxybutyate-co-hydroxyvalerate (PHBV) local antibiotic delivery system: In vivo effectiveness and biocompatibility in the treatment of implant-related experimental osteomyelitis. J. Biomed. Mater. Res..

[38] Huang W., Shi X., Ren L., Du C., Wang Y. (2010). PHBV microspheres–PLGA matrix composite scaffold for bone tissue engineering. Biomaterials.

[39] Zhao K., Deng Y., Chen J.C., Chen G.Q. (2003). Polyhydroxyalkanoate (PHA) scaffolds with good mechanical properties and biocompatibility. Biomaterials.

[40] Lizarraga-Valderrama L.R., Nigmatullin R., Taylor C., Haycock J.W., Claeyssens F., Knowles J.C., Roy I. (2015). Nerve tissue engineering using blends of poly(3-hydroxyalkanoates) for peripheral nerve regeneration. Eng. Life Sci..

[41] Rehm B.H. (2003). Polyester synthases: Natural catalysts for plastics. Biochem. J..

[42] Lee W.H., Azizan M.N.M., Sudesh K. (2004). Effect of culture conditions of poly(3-hydroxybuyrate-co-4-hydroxybutyrate) synthesized by *Commamonas acidovorans*. Polym. Degrad. Stab..

[43] Ciesielski S., Możejko J., Przybyłek G. (2010). The influence of nitrogen limitation on mcl-PHA synthesis by two newly isolated strains of *Pseudomonas* sp.. J. Ind. Microbiol. Biotechnol..

[44] Freches A., Lemos P.C. (2017). Microbial selection strategies for polyhydroxyalkanoates production from crude glycerol: Effect of OLR and cycle length. New Biotechnol..

[45] Surendran A., Lakshmanan M., Chee J.Y., Sulaiman A.M., Van Thuoc D., Sudesh K. (2020). Can Polyhydroxyalkanoates Be Produced Efficiently From Waste Plant and Animal Oils?. Front. Bioeng. Biotechnol..

[46] Das S., Majumder A., Shukla V., Suhazsini P., Radha P. (2018). Biosynthesis of Poly(3-hydroxybutyrate) from Cheese Whey by *Bacillus megaterium* NCIM 5472. J. Polym. Environ..

[47] Obruca S., Marova I., Melusova S., Mravcova L. (2011). Production of polyhydroxyalkanoates from cheese whey employing *Bacillus megaterium* CCM 2037. Ann. Microbiol..

[48] Kucera D., Pernicová I., Kovalcik A., Koller M., Mullerova L., Sedlacek P., Mravec F., Nebesarova J., Kalina M., Marova I. (2018). Characterization of the promising poly(3-hydroxybutyrate) producing halophilic bacterium *Halomonas halophila*. Bioresour. Technol..

[49] Bustamante D., Segarra S., Tortajada M., Ramon D., del Cerro C., Prieto M.A., Iglesias J.R., Rojas A. (2019). In silico prospection of microorganisms to produce polyhydroxyalkanoate from whey: *Caulobacter segnis* DSM 29236 as a suitable industrial strain. Microbial. Biotechnol..

[50] Pantazaki A.A., Papaneophytou C.P., Pritsa A.G., Liakopoulou-Kyriakide M., Kyriakidis D.A. (2009). Production of polyhydroxyalkanoates from whey by *Thermus thermophilus* HB8. Process. Biochem..

[51] de Andrade C.S., Nascimento V.M., Cortez Vega W.R., Fakhouri F.M., Silva L.F., Gomez J.B.G., Fonseca G. (2017). Exploiting Cheese Whey as Co-substrate for Polyhydroxyalkanoates Synthesis from *Burkholderia sacchari* and as Raw Material for the Development of Biofilms. Waste Biomass Valorization.

[52] Suhazsini P., Keshav R., Narayanan S., Chaudhuri A., Radha P. (2020). A Study on the Synthesis of Poly (3-hydroxybutyrate-co-3-hydroxyvalerate) by *Bacillus megaterium* Utilizing Cheese Whey Permeate. J. Polym. Environ..

[53] Pais J., Serafin L.S., Freitas F., Reis M.A.M. (2016). Conversion of cheese whey into poly(3-hydroxybutyrate-co-3-hydroxyvalerate) by *Haloferax mediterranei*. New Biotechnol..

[54] Singh A.K., Mallick N. (2009). Exploitation of inexpensive substrates for production of a novel SCL–LCL-PHA co-polymer by *Pseudomonas aeruginosa* MTCC 7925. Ind. Microbiol. Biotechnol..

[55] de Paula F.C., Kakazu S., de Paula C.B.C., Gomez J.G.C., Contiero J. (2016). Polyhydroxyalkanoate production from crude glycerol by newly isolated *Pandoraea* sp.. J. King. Saud. Univ..

[56] de Paula F.C., Kakazu S., de Paula C.B.C., de Almeida A.F., Gomez J.G.C., Contiero J. (2019). *Burkholderia glumae* MA13: A newly isolated bacterial strain suitable for polyhydroxyalkanoate production from crude glycerol. Biocatal. Agric. Biotechnol..

[57] Gahlawat G., Soni S.K. (2019). Study on Sustainable Recovery and Extraction of Polyhydroxyalkanoates (PHAs) Produced by *Cupriavidus necator* Using Waste Glycerol for Medical Applications. Chem. Biochem. Eng. Q..

[58] Ribeiro P.L.L., da Silva A.C.M.S., Filho J.A.M., Druzian J.I. (2015). Impact of different by-products from the biodiesel industry and bacterial strains on the production, composition, and properties of novel polyhydroxyalkanoates containing achiral building blocks. Ind. Crops Prod..

[59] Poblete-Castro I., Binger D., Oehlert R., Rohde M. (2014). Comparison of mcl-Poly(3-hydroxyalkanoates) synthesis by different *Pseudomonas putida* strains from crude glycerol: Citrate accumulates at high titer under PHA-producing conditions. BMC Biotechnol..

[60] Kourmentza C., Araujo D., Sevrin C., Roma-Rodriques C., Ferreira J.L., Freitas F., Dionisio M., Baptista P.V., Fernandes A.R., Grandfils C. (2019). Occurrence of non-toxic bioemulsifiers during polyhydroxyalkanoate production by *Pseudomonas* strains valorizing crude glycerol by-product. Bioresour. Technol..

[61] Pereira J.R., Araújo D., Marques A.C., Neves L.A., Grandfils C., Sevrin C., Alves V.D., Fortunato E., Reis M.A.M., Freitas F. (2019). Demonstration of the adhesive properties of the medium-chain-length polyhydroxyalkanoate produced by *Pseudomonas chlororaphis* subsp. *aurantiaca* from glycerol. Int. J. Biol. Macromol..

[62] Muangwong A., Boontip T., Pachimsawat J., Napathorn S.C. (2016). Medium chain length polyhydroxyalkanoates consisting primarily of unsaturated 3-hydroxy-5-cis-dodecanoate synthesized by newly isolated bacteria using crude glycerol. Microb. Cell. Fact..

[63] Desouky S.E.S., Abdel-Rahman M.A., Azab M.S., Esmael M.E. (2017). Batch and fed-batch production of polyhydroxyalkanoates from sugarcane molasses by *Bacillus flexus* Azu-A2. JIPBS.

[64] Kulpreecha S., Boonruangthavorn A., Meksiriporn B., Thongchul N. (2009). Inexpensive fed-batch cultivation for high poly(3-hydroxybutyrate) production by a new isolate of *Bacillus megaterium*. J. Biosci. Bioeng..

[65] Geethu M., Vrundha R., Raja S., Raghu H., Chandrashekar H.R., Divyashree M.S. (2019). Improvement of the Production and Characterisation of Polyhydroxyalkanoate by *Bacillus endophyticus* Using Inexpensive Carbon Feedstock. J. Polym. Environ..

[66] Dalsasso R.R., Pavan F.A., Bordignon S.E., de Aragão G.M.F., Poletto P. (2019). Polyhydroxybutyrate (PHB) production by *Cupriavidus necator* from sugarcane vinasse and molasses as mixed substrate. Process. Biochem..

[67] Mohammed S., Panda A.A., Ray L. (2019). An investigation for recovery of polyhydroxyalkanoates (PHA) from *Bacillus* sp. BPPI-14 and *Bacillus* sp. BPPI-19 isolated from plastic waste landfill. Int. J. Biol. Macromol..

[68] Rathika R., Janaki V., Shanthi K., Kamala-Kannan S. (2019). Bioconversion of agro-industrial effluents for polyhydroxyalkanoates production using *Bacillus subtilis* RS1. Int. J. Environ. Sci. Tech..

[69] Hassan M.A., Bakhiet E.K., Hussein H.R., Ali S.G. (2018). Statistical optimization studies for polyhydroxybutyrate (PHB) production by novel *Bacillus subtilis* using agricultural and industrial wastes. Int. J. Sci. Environ. Technol..

[70] Hassan E.A., Abd-Alla M.H., Zohri A.N.A., Ragaey M.M., Ali S.M. (2019). Production of butanol and polyhydroxyalkanoate from industrial waste by *Clostridium beijerinckii* ASU10. Int. J. Energy Res..

[71] Kourmentza C., Costa J., Azevedo Z., Servin C., Grandfils C., De Freitas V., Reis M.A.M. (2018). *Burkholderia thailandensis* as a microbial cell factory for the bioconversion of used cooking oil to polyhydroxyalkanoates and rhamnolipids. Bioresour. Technol..

[72] Thuoc D.V., Anh V.T.M. (2021). Bioconversion of Crude Fish Oil Into Poly-3-hydroxybutyrate by *Ralstonia* sp. M91. Appl. Biochem. Microbiol..

[73] Pernicova I., Kucera D., Nebesarova J., Kalina M., Novackova I., Koller M., Obruca S. (2019). Production of polyhydroxyalkanoates on waste frying oil employing selected *Halomonas* strain. Bioresour. Technol..

[74] Sangkharak K., Prasertsan P. (2013). The production of polyhydroxyalkanoate by *Bacillus licheniformis* using sequential mutagenesis and optimization. Biotechnol. Bioprocess Eng..

[75] Kumar P., Kim B.S. (2019). *Paracoccus* sp. Strain LL1 as a Single Cell Factory for the Conversion of Waste Cooking Oil to Polyhydroxyalkanoates and Carotenoids. Appl. Food Biotechnol..

[76] Obruca S., Petrik S., Benesova P., Svoboda Z., Eremka L., Marova I. (2014). Utilization of oil extracted from spent coffee grounds for sustainable production of polyhydroxyalkanoates. Appl. Microbiol. Biotechnol..

[77] Omar F.N., Rahman N.A., Hafid H.S., Mumtaz T., Yee P.L., Hassan M.A. (2011). Utilization of kitchen waste for the production of green thermoplastic polyhydroxybutyrate (PHB) by *Cupriavidus necator* CCGUG 52238. Afr. J. Microbiol. Res..

[78] Kovalcik A., Pernicova I., Obruca S., Szotkowski M., Enev V., Kalina M., Marova I. (2020). Grape winery waste as a promising feedstock for the production of polyhydroxyalkanoates and other value-added products. Food Bioprod. Process..

[79] Sukruansuwan V., Napathorn S.C. (2018). Use of agro industrial residue from the canned pineapple industry for polyhydroxybutyrate production by *Cupriavidus necator* strain A 04. Biotechnol. Biofuels.

[80] Kovalcika A., Kucera D., Matouskova P., Pernicova I., Obruca S., Kalina M., Enev V., Marova I. (2018). Influence of removal of microbial inhibitors on PHA production from spent coffee grounds employing *Halomonas halophila*. J. Environ. Chem. Eng..

[81] Obruca S., Benesova P., Petrika S., Oborna J., Prikryl R., Marova I. (2014). Production of polyhydroxyalkanoates using hydrolysate of spent coffee grounds. Process. Biochem..

[82] Rebocho A.T., Pereira J.R., Freitas F., Neves L.A., Alves C.D., Sevrin C., Grandfils C., Reis M.A.M. (2019). Production of Medium-Chain Length Polyhydroxyalkanoates by *Pseudomonas citronellolis* Grown in Apple Pulp Waste. Appl. Food Biotechnol..

[83] Mollea C., Marmo L., Bosco F., Mazzalupo I. (2019). Valorisation of cheese whey, a by-product from the dairy industry. Food Industry.

[84] Povolo S., Toffano P., Basaglia M., Casella S. (2010). Polyhydroxyalkanoates production by engineered *Cupriavidus necator* from waste material containing lactose. Bioresour. Technol..

[85] Berwig K.H., Baldasso C., Dettmer A. (2016). Production and characterization of poly(3-hydroxybutyrate) generated by *Alcaligenes latus* using lactose and whey after acid protein precipitation process. Bioresour. Technol..

[86] Yellore V., Desai A. (1998). Production of poly-3-hydroxybutyrate from lactose and whey by *Methylobacterium* sp. ZP24. Lett. Appl. Microbiol..

[87] Koller M. (2015). Recycling of waste streams of the biotechnological poly(hydroxyalkanoate) production by *Haloferax mediterranei* on whey. Int. J. Polym. Sci..

[88] Da Silva G.P., Mack M., Contiero J. (2009). Glycerol: A promising and abundant carbon source for industrial microbiology. Biotechnol. Adv..

[89] Sharma P.K., Fu J., Spicer V., Krokhin O.V., Cicek N., Sparling R., Levin D.B. (2016). Global changes in the proteome of *Cupriavidus necator* H16 during poly-(3-hydroxybutyrate) synthesis from various biodiesel by-product substrates. AMB Express.

[90] Zhu C., Chiu S., Nakas J., Nomura C. (2013). Bioplastics from waste glycerol derived from biodiesel industry. J. Appl. Polym. Sci..

[91] Możejko-Ciesielska J., Pokój T. (2018). Exploring nutrient limitation for polyhydroxyalkanoates synthesis by newly isolated strains of *Aeromonas* sp. using biodiesel-derived glycerol as a substrate. Peer J..

[92] Bhattacharya B., Dubey S., Singh P., Shrivastava A., Mishra S. (2016). Biodegradable Polymeric Substances Produced by a Marine Bacterium from a Surplus Stream of the Biodiesel Industry. Bioengineering.

[93] Kumar P., Ray S., Patel S.K.S., Lee J.K., Kalia V.C. (2015). Bioconversion of crude glycerol to polyhydroxyalkanoate by *Bacillus thuringiensis* under non-limiting nitrogen conditions. Int. J. Biol. Macromol..

[94] Canadas R.F., Cavalheiro J.M.B.T., Guerreiro J.D.T., de Almeida M.C.M.D., Pollet E., da Silva C.L., da Fonseca M.M.R., Ferreira F.C. (2014). Polyhydroxyalkanoates: Waste glycerol upgrade into electrospun fibrous scaffolds for stem cells culture. Int. J. Biol. Macromol..

[95] Ibrahim M.H., Steinbüchel S. (2010). *Zobellella denitrificans* strain MW1, a newly isolated bacterium suitable for poly(3-hydroxybutyrate) production from glycerol. J. Appl. Microbiol..

[96] Pavan F.A., Junqueira T.L., Watanabe M.D.B., Bonomi A., Quines L.K., Schmidell W., de Aragao G.M.F. (2019). Economic analysis of polyhydroxybutyrate production by *Cupriavidus necator* using different routes for product recovery. Biochem. Eng. J..

[97] Qureshi N., Lolas A., Blaschek H.P. (2001). Soy molasses as fermentation substrate for production of butanol using *Clostridium beijerinckii* BA101. J. Ind. Microbiol. Biotechnol..

[98] Ciesielski S., Możejko-Ciesielska J., Pisutpaisal N. (2015). Plant oils as promising substrates for polyhydroxyalkanoates production. J. Clean. Prod..

[99] Chen G.Q., Jiang X.R. (2018). Next generation industrial biotechnology based on extremophilic bacteria. Curr. Opin. Biotechnol..

[100] Ruiz C., Kenny S.T., Narancic T., Babu R., Connor K.O. (2019). Conversion of waste cooking oil into medium chain polyhydroxyalkanoates in a high cell density fermentation. J. Biotechnol..

[101] Follonier S., Goyder M.S., Silvestri A.C., Crelier S., Kalman F., Riesen R., Zinn M. (2014). Fruit pomace and waste frying oil as sustainable resources for the bioproduction of medium-chain-length polyhydroxyalkanoates. Int. J. Biol. Macromol..

[102] Follonier S., Riesen R., Zinn M. (2015). Pilot-scale production offunctionalized mcl-PHA from grape pomace supplemented with fatty acids. Chem. Biochem. Eng. Q..

[103] Gowda V., Shivakumar S. (2014). Agrowaste-based Polyhydroxyalkanoate (PHA) Production using Hydrolytic Potential of *Bacillus thuringiensis* IAM 12077. Braz. Arch. Biol. Technol..

[104] Pleissner D., Lam W.C., Han W., Lau K.Y., Cheung L.C., Lee M.W., Lei H.M., Lo K.Y., Ng W.Y., Sun Z. (2014). Fermentative Polyhydroxybutyrate Production from a Novel Feedstock Derived from Bakery Waste. J. Biomed. Biotechnol..

[105] Sabapathy P.C., Devaraj S., Parthipan A., Kathirvel P. (2019). Polyhydroxyalkanoate production from statistically optimized media using rice mill effluent as sustainable substrate with an analysis on the biopolymer’s degradation potential. Int. J. Biol. Macromol..

[106] Amini M., Yousefi-Massumabad H., Younesi H., Abyar H., Bahramifar N. (2019). Production of the polyhydroxyalkanoate biopolymer by *Cupriavidus necator* using beer brewery wastewater containing maltose as a primary carbon source. J. Environ. Chem. Eng..

[107] Al-Battashi H., Annamalai N., Al-Kindi S., Nair N.S., Al-Bahry S., Verma J.P., Sivakumar N. (2019). Production of bioplastic (poly-3-hydroxybutyrate) using waste paper as a feedstock: Optimization of enzymatic hydrolysis and fermentation employing *Burkholderia sacchari*. J. Clean. Prod..

[108] Birgham C.J., Riedel S.L. (2019). The potential of polyhydroxyalkanoate production from food wastes (Review). Appl. Food Biotechnol..

[109] Pais J., Farinha I., Freitas F., Serafim L.S., Martínez V., Martínez J.C., Arévalo-Rodríguez M., Prieto M.A., Reis M.A.M. (2014). Improvement on the yield of polyhydroxyalkanoates production from cheese whey by a recombinant *Escherichia coli* strain using the proton suicide methodology. Enzym. Microb. Technol..

[110] Saranya V., Shenbagarathai R. (2011). Production and characterization of PHA from recombinant *E. coli* harbouring phaC1 gene of indigenous *Pseudomonas* sp. LDC-5 using molasses. Braz. J. Microbiol..

[111] Obruca S., Snajdar O., Svoboda Z., Marova I. (2013). Application of random mutagenesis to enhance the production of polyhydroxyalkanoates by *Cupriavidus necator* H16 on waste frying oil. World J. Microbiol. Biotechnol..

[112] Annamalai N., Sivakumar N. (2016). Production of polyhydroxybutyrate from wheat bran hydrolysateusing *Ralstonia eutropha* through microbial fermentation. J. Biotechnol..

[113] Riedel S.L., Jahns S., Koeniga S., Bocka M.C.E., Brighamc C.J., Bader J., Stahl U. (2015). Polyhydroxyalkanoates production with *Ralstonia eutropha* from low quality waste animal fats. J. Biotechnol..

[114] Kamilah H., Tsuge T., Yang T., Sudesh K. (2013). Waste cooking oil as substrate for biosynthesis of poly(3-hydroxybutyrate) and poly(3-hydroxybutyrate-co-3-hydroxyhexanoate): Turning waste into a value-added product. Malays. J. Microbiol..

[115] Bhatia S.K., Kim J.H., Kim M.S., Kim J., Hong J.W., Hong J.G., Kim H.J., Jeon J.M., Kim S.H., Ahn J. (2018). Production of (3-hydroxybutyrate-co-3-hydroxyhexanoate) copolymer from coffee waste oil using engineered *Ralstonia eutropha*. Bioproc. Biosyst. Eng..

[116] Thinagaran L., Sudesh K. (2017). Evaluation of Sludge Palm Oil as Feedstock and Development of Efficient Method for its Utilization to Produce Polyhydroxyalkanoate. Waste Biomass Valorization.

[117] Borrero-de Acuña J.M., Aravena-Carrasco C., Carla Gutiérrez-Urrutia I., Duchens D., Poblete-Castro I. (2019). Enhanced synthesis of medium-chain-length Poly(3-hydroxyalkanoates) by inactivating the tricarboxylate transport system of *Pseudomonas putida* KT2440 and process development using waste vegetable oil. Process Biochem..

[118] Sharma P.K., Munir R., Riffat de Kievit T., Levin D.B. (2017). Synthesis of Polyhydroxyalkanoates (PHAs) from vegetable oils and free fatty acids by wild-type and mutant strains of *Pseudomonas chlororaphis*. Can. J. Microbiol..

[119] de Paula F.C., de Paula C.B.C., Gomez J.G.C., Steinbuchel A., Contiero J. (2017). Poly(3 hydroxybutyrate-co-3-hydroxyvalerate) Production from Biodiesel By-Product and Propionic Acid by Mutant Strains of *Pandoraea* sp.. Biotechnol. Prog..

[120] Li M., Wilkins M.R. (2020). Recent advances in polyhydroxyalkanoate production: Feedstocks, strains and process developments. Int. J. Biol. Macromol..

[121] Sun Z., Ramsay J.A., Guay M., Ramsay B.A. (2007). Fermentation process development for the production of medium-chain-length poly-3-hyroxyalkanoates. Appl. Microbiol. Biotechnol..

[122] Li D., Lv L., Chen J.C., Chen G.Q. (2017). Controlling microbial PHB synthesis via CRISPRi. Appl. Microbiol. Biotechnol..

